# The Induction and Roles Played by Phi Thickenings in Orchid Roots

**DOI:** 10.3390/plants8120574

**Published:** 2019-12-05

**Authors:** Nurul A. Idris, David A. Collings

**Affiliations:** 1 Faculty of Science and Marine Environment, Universiti Malaysia Terengganu, Kuala Terengganu 21030, Terengganu, Malaysia; nurul.aliaa@umt.edu.my; 2 School of Biological Sciences, University of Canterbury, Private Bag 4800, Christchurch 8140, New Zealand; 3 School of Environmental and Life Sciences, The University of Newcastle, Callaghan NSW 2308, Australia

**Keywords:** *Miltoniopsis*, orchid, phi thickening, root morphology, secondary cell wall, water stress

## Abstract

Phi thickenings are specialised secondary wall thickenings present in the root cortex of many plant species, including both angiosperms and gymnosperms. While environmental stresses induce phi thickenings, their role(s) in the root remain unclear. Suggested functions include regulation of transport through the apoplast in a manner similar to the Casparian strip, limiting fungal infections, and providing mechanical support to the root. We investigated phi thickening induction and function in *Miltoniopsis* sp., an epiphytic orchid. As movement of a fluorescent tracer through the apoplast was not blocked by phi thickenings, and as phi thickenings developed in the roots of sterile cultures in the absence of fungus and did not prevent fungal colonisation of cortical cells, the phi thickenings in *Miltoniopsis* did not function as a barrier. Phi thickenings, absent in roots grown on agar, remained absent when plants were transplanted to moist soil, but were induced when plants were transplanted to well-drained media, and by the application of water stress. We suggest that it is likely that phi thickenings stabilise to the root during water stress. Nevertheless, the varied phi thickening induction responses present in different plant species suggest that the phi thickenings may play multiple adaptive roles depending on species.

## 1. Introduction

Phi thickenings are specialised secondary cell walls that form bands around the cortical cells in roots that normally only have a primary cell wall. Because these isolated bands often appear near circular in cross section, containing a thin primary cell wall running down their centre, they resemble and are named for the Greek letter phi (ϕ). However, the term is now used to encompass all patterned bands of secondary thickenings in the root cortex [1]. Initially observed in gymnosperms [2], these structures have been described in more than 100 plant species from 16 families, including both monocots and eudicots (reviewed in [1,3,4]). As phi thickenings are present in diverse taxa, the basic capacity to generate a phi thickening has either evolved multiple times during the evolution, or it has been conserved for a very long period of time. In either case, this conservation indicates the fundamental importance of phi thickenings for plant growth. Furthermore, the wide distribution of phi thickenings suggests that the formation of phi thickenings can be a deliberate response mechanism by plants to stress, and that they can be functionally important for root growth. The roles played by phi thickenings, and why such a wide variety of plants develop them, remain unclear, although three possible roles for phi thickenings have been proposed. These are to regulate solute uptake, to defend against penetration by fungal hyphae, and to mechanically stabilise the root, either as a response to environmental stresses or to allow the root to overcome high soil impedance. These roles would not be mutually exclusive, and it is possible that different plants might develop phi thickenings for different reasons.

Phi thickenings have been proposed to act a physical barrier against hyphal penetration. Following fungal invasions in yellow birch (*Betula alleghaniensis*) [5] and *Dryas integrifolia* [6], the plants form a symbiotic relationship with ectomycorrhiza in the root cortex. In yellow birch, the fungus penetrates the epidermis into the cortex, but seems to be obstructed from entering further into the root by phi thickenings present in the second cortical layer [5], while in *Dryas*, a double layer of phi thickenings in the radial cell walls of inner cortical cells limits fungal penetration beyond the cortex [6].

Regulation of solute uptake was one of the earliest functions suggested for phi thickenings, due to their apparent similarity to the Casparian band in the endodermis [7,8]. In *Pelargonium*, however, the ultrastructure of phi thickenings differs significantly from the Casparian band. The phi thickenings lignify early in root development, and the plasma membrane does not adhere strongly to the cell wall of the phi thickening, two features that are unlike what occurs with the Casparian band [9]. Studies have also tested the permeability of the phi thickenings using fluorescent tracers. These experiments showed that phi thickenings in apple and geranium roots did not block apoplastic flow of the small fluorescent dye calcofluor white [10], and that thickenings in maize roots failed to block the flow of berberine hemisulfate [11], even though both these dyes were blocked at Casparian strips. These observations do not, however, rule out a role for phi thickenings in the regulation of solute uptake in other plant species. In broccoli, for example, phi thickenings induced by salinity reduced the uptake of lanthanum, an apoplastic transport tracer, indicating a possible role for the phi cells as a barrier controlling the movement of ions during salt stress [12].

The induction of phi thickening formation as a response to environmental stresses and/or changes in growth conditions also suggests that the new, lignified secondary cell walls might be able to mechanically support cortical cells [1], a suggestion regarding phi thickenings initially made by Van Tieghem [13]. In cherry (*Prunus avium*), roots grown in culture on MS-based agar medium lacked phi thickenings, but these developed when the plants were transplanted to soil [14], with similar changes also observed in *Prunus* crosses [15]. Similarly, phi thickenings were reported to be induced in broccoli (*Brassica oleracea*) by salinity [12,16]. Furthermore, in both *B. oleracea* and *B. napus*, phi thickenings could be induced by both salt- and sucrose-dependent water stress, but in a variety-dependent manner, with some cultivars seemingly lacking phi thickening induction in the primary root [17]. Phi thickening-like structures were also induced by salt in radish (*Raphanus sativus*) roots [18], while phi thickening induction in loquat roots resulted from water stress or drought [19]. Furthermore, in maize (*Zea mays*) grown in municipal solid-waste slag waste, the phi thickenings induced in the rhizodermis have been suggested to play a mechanical role to overcome the high soil impedance [11]. Similarly, the induction of phi thickenings in gymnosperms correlated with the degree of freezing to which they were exposed, although the structures were constitutively expressed to a low degree in all the trees investigated [20].

We have previously investigated the formation of phi thickenings in the roots of the South American epiphytic orchid *Miltoniopsis*, and have shown that these secondary cell walls are lignified but not suberised [21]. Phi thickenings have also been reported in a range of other orchid species [22]. Moreover, many epiphytic orchids whose roots often grow exposed to the air show extensive secondary thickenings of their cortical cell walls that are referred to as a “pseudo-velamen” [23], which might also be described as elaborate phi thickenings. It may be important that while these structures are common in epiphytic orchids, they are absent or rare in terrestrial orchids whose roots grow in soil [24]. In this study, we explored phi thickening function in *Miltoniopsis.* We demonstrate that the thickenings are independent of fungal infection, and do not block the uptake of apoplastic tracers. However, phi thickenings can be deliberately induced by water stress, and may function in the stabilisation of the root cortex.

## 2. Results

### 2.1. Miltoniopsis Orchid Roots Can Contain Phi Thickenings

**Figure 1:**
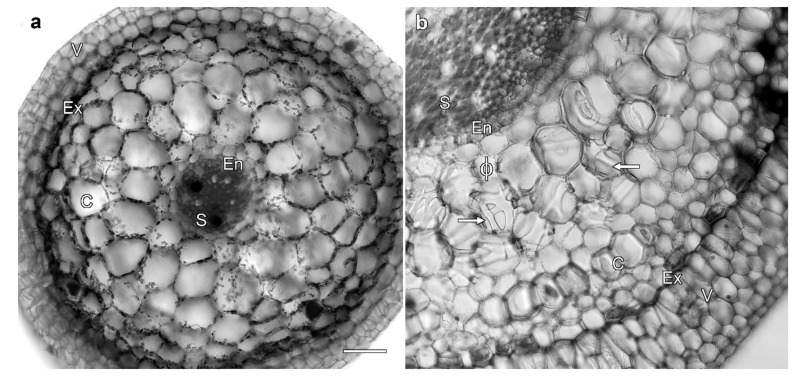
Root morphology depends on growth conditions. Cross-sections imaged with transmitted light of *Miltoniopsis* roots grown in vitro (**a**) and in soil (**b**). Both roots consist of (in order from the exterior of the root) a velamen layer (V), exodermis (Ex), cortex (C), endodermis (En), and stele (S). Only the much larger soil-grown roots (**b**) had a well-developed velamen, and contained phi thickenings with these viewed both in cross-section (ϕ) and as bands that loop across the cell ends (arrows). Bar represents 100 μm for both images.

Roots of epiphytic *Miltoniopsis* orchids show morphological changes in response to culture conditions. When grown on agar, they did not develop a fully mature velamen layer (Figure 1a), but when transferred to soil, the plants developed much thicker roots that contained a strongly-developed velamen (Figure 1b, shown at the same magnification as Figure 1a). Furthermore, as plants grown on agar lacked phi thickenings, and as these structures were present in the cortex of soil-grown plants (Figure 1b) viewed both in section (ϕ) and at the ends of cells (arrows), it was possible to manipulate the development of phi thickenings through the use of culture conditions. This allowed the investigation of the external factors that induce phi thickenings and, consequently, their function. 

**Figure 2:**
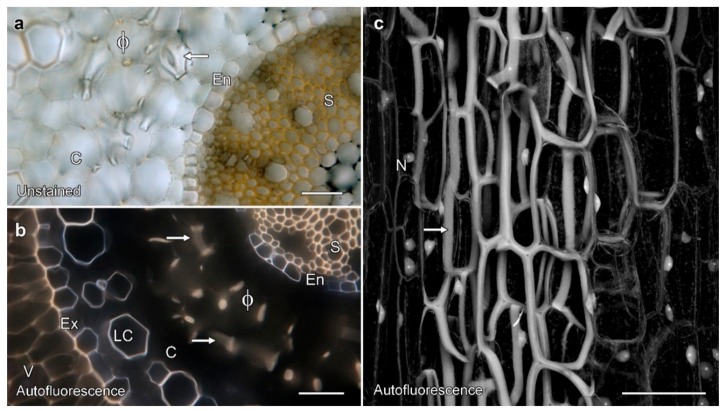
Phi thickenings are lignified secondary cell walls that frame cortical cells, and were visible in unstained cross-sections imaged by bright-field microscopy (**a**). Autofluorescent thickenings were also visible in cross sections with UV excitation and a long-pass filter (**b**), and in an unstained longitudinal section imaged by confocal microscopy using 405 nm excitation (**c**). (**a**) Phi thickenings in cross-section (ϕ) and at the ends of cells (arrow) were visible in the root cortex of unstained samples. (**b**) Lignified phi thickenings were autofluorescent. (**c**) A maximum projection image of 66 lignin autofluorescence images collected by confocal microscopy over a depth of 98 μm. The phi thickenings form a cage encircling the cortical cells, so that a section through the middle of a cell showed thickenings would appear like the Greek letter ϕ, and sections containing cell ends would give bands (arrows in (**a**) and (**b**)). V, velamen; Ex, exodermis; LC, lignified cortical cell; Ex, exodermis; S, stele; N, autofluorescent nucleus. Bar represents 100 μm in each image.

We characterised phi thickening structure in unstained material using various modes of transmitted light and fluorescence microscopy (Figure 2). Cross-sections through roots grown in soil showed bands of secondary cell walls in the root cortex, visible with both transmitted light (Figure 2a), and with lignin autofluorescence visible with UV excitation using conventional fluorescence microscopy (Figure 2b). Lignification and autofluorescence were also present in the central stele (S), the endodermis (En), outer cortical cells (LC), exodermis (Ex), and velamen (V), although the differences in fluorescence colour suggest chemical differences in the walls of the different cells. By confocal microscopy, lignin autofluorescence was readily detectable in phi thickenings (Figure 2c), with maximum projections of optical series emphasising the three dimensionality of the thickening network around the cortical cells, and the fact that the thickenings form a box that encircles the cortical cells. Labelling also revealed autofluorescent nuclei (N) that, in cross-section, could be distinguished from phi thickenings through a comparison of shape and their depth.

### 2.2. Permeability Tests on Phi Thickenings

**Figure 3:**
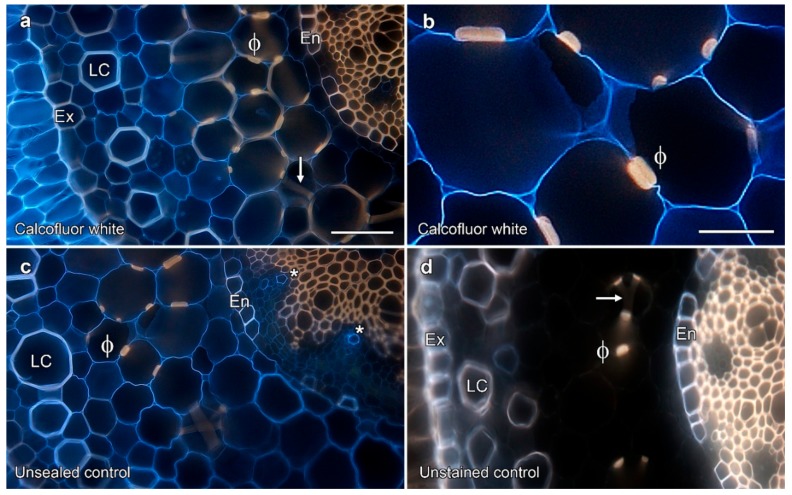
Phi thickenings did not block the flow of the tracer dye calcofluor white. (**a**–**d**) Ultraviolet excitation of lignin and calcofluor white. (**a**) Apoplastic flow of calcofluor white, visible as blue fluorescence, was not blocked by phi thickenings (arrow and ϕ), but was restricted by the endodermis (En). Yellow autofluorescence derives from the lignified tissue. (**b**) Higher magnification image of calcofluor white permeability through phi thickenings (ϕ). (**c**) Blue fluorescence from calcofluor white was present in the xylem (asterisks) in the unsealed control. (**c**) Unstained control showing lignin autofluorescence. LC, lignified cortical cell; Ex, exodermis. Bar in (**a**) represents 100 μm for (**a**), (**c**), and (**d**); bar in (**b**) represents 50 μm.

The ability for small solutes to pass apoplastically through the cell walls thickened by phi thickenings was tested with small, membrane-impermeant fluorescent tracers. Diffusion of calcofluor white was not blocked by phi thickenings in apple and geranium roots, but was blocked by the endodermis [10]. Similar results were obtained with calcofluor in *Miltoniopsis* roots, with penetration of calcofluor white into cell walls inside phi thickenings (Figure 3a,b). Calcofluor diffusion was, however, blocked by the endodermal layer and blue fluorescence was not visible inside the endodermis or with the xylem vessels. This lack of labelling in the vascular tissue demonstrated that labelling seen inside the phi thickenings was unlikely to be due to dye diffusion after sectioning. In contrast, control samples in which the ends of the root had not been glued showed blue calcofluor fluorescence inside the endodermis that was associated with the xylem vessels, as well as cell wall fluorescence from throughout the cortex (Figure 3c).

### 2.3. Fungal Infections Are Not Required to Induce Phi Thickenings

**Figure 4:**
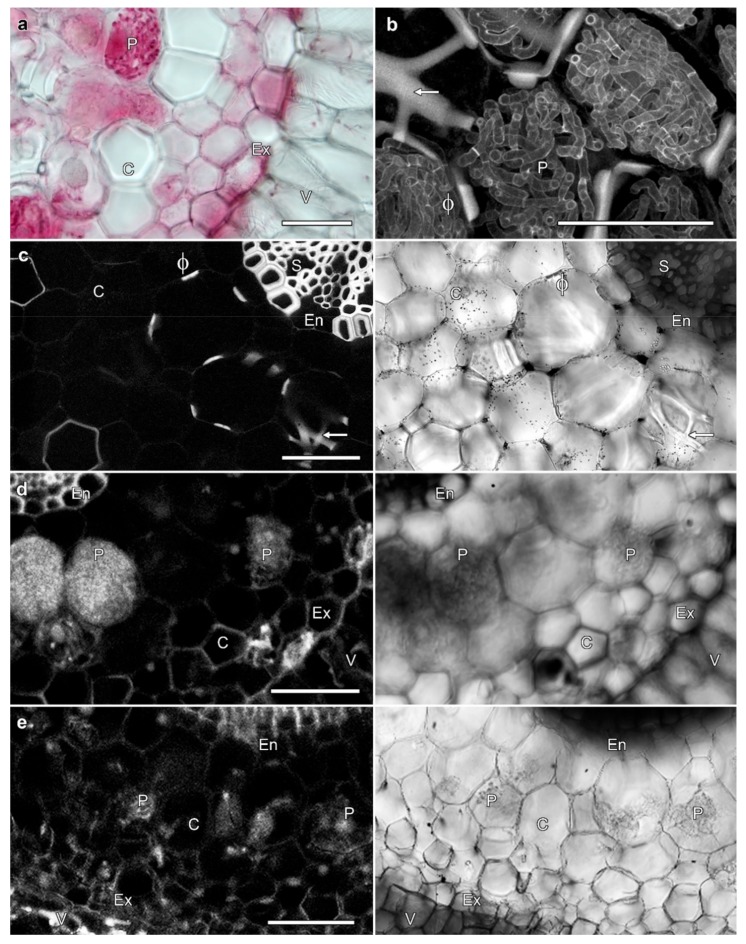
Phi thickening formation is independent of fungal mycorrhizae. (**a**,**b**) Lignin and fungal labelling, with lactofuchsin staining viewed by transmitted light (**a**), and lignin and fungal autofluorescence viewed by confocal microscopy (**b**). Dense hyphal coils (pelotons, P) were present in cortical cells containing phi thickenings (arrow). (**c**) Phi thickenings (ϕ and arrow) were induced in roots grown in bark in sterile conditions, and in the absence of orchid mycorrhizae. Images are a single confocal optical section showing lignin autofluorescence (left), and concurrent transmitted light image (right). (**d**) Extensive fungal pelotons (P) were present in the roots of sterile, agar-grown plants 4 weeks after transfer to un-sterilised but well-watered bark in the greenhouse. No phi thickenings were present. Images are transmitted light (left) and confocal imaging of lignin and fungal autofluorescence (right). (**e**) Fungal pelotons (P) were present in sterile, agar-grown roots 14 days after fungal reinfection. Images are transmitted light (left) confocal imaging of lignin and fungal autofluorescence (right). No phi thickenings were present. V, velamen; Ex, exodermis; LC, lignified outer cortical cells; C, cortex; En, endodermis. Bars represent 100 μm.

No correlation was observed between the presence and absence of phi thickenings, and the presence or absence of mycorrhizae. The roots of *Miltoniopsis* plantlets grown in sterile conditions on agar lack phi thickenings with these never observed in any roots, but the roots of plants grown in regular orchid mix typically contained phi thickenings (Figure 1a,b). Phi thickenings could occur in cells in which orchid mycorrhizae had formed pelotons (Figure 4b, arrow). To determine whether the presence of fungal association in the roots was required for phi thickening development, we transplanted sterile plants to orchid mix under sterile conditions, and phi thickenings were observed to develop in roots within 16 weeks (Figure 4c, arrow). If sterile plants were transferred to un-sterilised and well-watered bark in the greenhouse, fungal pelotons rapidly developed, but phi thickenings were absent (Figure 4d, P). And in the reverse experiment, a pure fungal culture isolated from *Miltoniopsis* roots was used to re-infect sterile roots grown on agar in a slanting double culture experiment. This fungal culture remains unidentified by molecular techniques, but its morphology and characteristics (right-angled branching hyphae adjacent to septa, and formation of moniloid cells on low nutrient agar) suggest that it is a *Tulasnella* species (John Dearnaley, University of Southern Queensland, *pers. comm*.). In these plants, fungal pelotons formed after 14 days of co-culture, but phi thickenings were not induced (Figure 4e, P). These experiments demonstrate that thickening induction in *Miltoniopsis* roots is independent of the infection of roots by orchid mycorrhizae.

### 2.4. Induction of Phi Thickenings

*Miltoniopsis* roots grown on agar lack phi thickenings, but these are induced by growth in regular orchid mix even under sterile conditions (Figure 4d). This ability to induce the development of phi thickenings in different conditions allowed the investigation of the external factors that may trigger development of phi thickenings and, consequently, their function. Other than this absence of phi thickenings, in vitro grown roots were smaller in diameter, contained chloroplasts in the outer cortex, and lacked a fully mature velamen. The induction and development of this complex velamen has been investigated [25]. To investigate how water stress can induce phi thickenings, *Miltoniopsis* plantlets which lacked phi thickenings were transplanted from in vitro culture to four different treatments that represented an increasing degree of compaction and the ability to retain water:

- Commercial orchid mix, made of big chunks of fir bark with finer pieces. This provided the least compaction and lowest water retention.
- Medium-sized pieces of fir bark.
- Medium-sized fir bark with pieces of sphagnum moss.
- Soil that consisted of fine fir bark that passed through a 6 mm diameter soil sieve. This mix provided the most compaction and best water retention.

**Figure 5:**
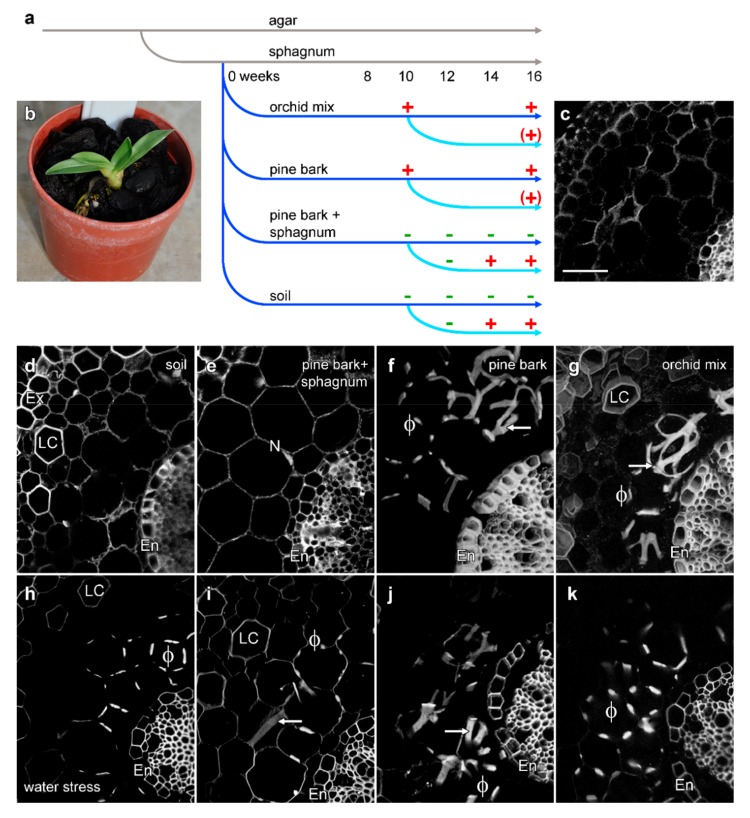
Water stress induced the formation of phi thickenings (arrows) in *Miltoniopsis* roots. (**a**) Diagrammatic representation of the transplantation of plants between different media, indicating the presence (**+**) or absence (**−**) of phi thickenings at different times. (**b**) Representative image of a *Miltoniopsis* plant growing in fir bark sphagnum mix. (**c**) Control plants grown on agar through the experiment did not generate phi thickenings, and had only a thin velamen. (**d**–**g**) Root cross sections from well-watered plants (watering twice per week) at 16 weeks. Confocal imaging of lignin autofluorescence showed no phi thickenings in plants grown in soil (**d**) or the fir bark sphagnum mix (**e**), but extensive phi thickenings (ϕ and arrows) were present in roots grown in fir bark (**f**) and orchid mix (**g**). (**h**–**k**) Root cross sections from water-stressed plants (watering once per week from week 10 onwards) at 16 weeks. Confocal imaging of lignin autofluorescence showed phi thickenings in all treatments, including soil (**h**) and the fir bark sphagnum mix (**i**), and in surviving plants grown in fir bark (**j**) and orchid mix (**k**). V, velamen; Ex, exodermis; LC, lignified cortical cell; En, endodermis. Bar in (**c**) represents 50 μm for all images.

Phi thickenings remained absent from agar-cultured roots for the entire 20 weeks of the experiment (Figure 5c). In transfer experiments, and after 10 weeks in which plants were watered twice per week, phi thickenings were observed in the two less compact and better drained substrates, the orchid mix and bark treatments (Figure 5f,g), but not in roots grown in either the soil or the bark/sphagnum moss mixture (Figure 5d,e). To investigate the effects of water stress, the watering frequency for a subset of plants in each soil treatment was then reduced to once a week. Water stress for 4 weeks induced phi thickenings in plants grown in both the soil and bark/sphagnum mixture (Figure 5h,i). Physically, the plants grown in both bark and orchid mix were more stressed during this reduced watering treatment with dry leaves and a lower production of new roots. After 4 weeks, 90% of the plants had shrivelled up and died, while plants grown in soil and the bark/sphagnum mix survived for up to 7 weeks before also drying out and dying.

## 3. Discussion

The ability to induce or diminish the presence of phi thickening through different growth conditions facilitates understanding of the functions that phi thickenings play within plant roots. We investigated the induction, and thus the functionality of phi thickenings in the roots of the *Miltoniopsis* orchids. Various stresses such as soil compaction, water stress, and fungal infection were tested, but the induction of phi thickenings was only achieved in a loosely compacted medium with relatively dry conditions, and fungal infection did not play a role. Permeability experiments showed that in this orchid, phi thickenings did not limit the apoplastic diffusion of tracers. Thus, in their induction by stress and their apparent function in stabilising the root, the phi thickenings in *Miltoniopsis* behave in a similar manner to phi thickenings in other angiosperms and gymnosperms [1].

### 3.1. Orchid Phi Thickenings Do Not Regulate Solute Uptake

Our data demonstrate that the proposed roles for phi thickenings of solute uptake regulation and the limitation of fungal infection do not occur in *Miltoniopsis* roots. Solute uptake regulation was one of the earliest functions suggested for phi thickenings due to their apparent similarity to the Casparian band in the endodermis. However, consistent with studies in several other plants [8,10,11], phi thickenings in *Miltoniopsis* do not block the apoplastic flow of calcofluor white. Furthermore, phi thickenings in *Miltoniopsis* [21] and other plants lack suberisation, and thus phi thickenings and the Casparian strip show major differences in structure [26]. The formation of a fully-functional Casparian strip that regulates water and ion uptake through the root, and that remains water-tight, is a complex process [27,28], but mutant *Arabidopsis* plants that lack suberin still have a functioning, lignified Casparian band [29]. Thus, it is lignification that is essential for Casparian strip functionality, although formation of a fully functional Casparian strip that remains water-tight also requires suberisation [30]. Some evidence does exist, however, for phi thickenings functioning to regulate ion movements within roots. For example, the phi cell layer can act as a barrier for movement of lanthanum ions when this was used as an apoplastic tracer in *Brassica oleracea* under salt stress [16], thus not entirely ruling out the function of phi thickenings in solute uptake regulation in other plants. Additional support for a possible role in regulating metal uptake include the observations of zinc-induction of phi thickenings and phi thickening-like structures in the roots of the metal hyper-accumulator *Thlaspi caerulescens* [31,32]. 

### 3.2. Orchid Phi Thickenings Do Not Regulate Fungal Penetration

While it has been suggested that phi thickenings can block the penetration of endomycorrhizal fungi in roots [5,6], we found no correlation between the presence of phi thickening and obstruction of fungal penetration, with phi thickenings formed in the cortex of the roots regardless of the presence or absence of a mycorrhizal interaction, and with fungal infections being independent of the presence or absence of phi thickenings. A similar lack of correlation was found in black alder [33].

### 3.3. Are Phi Thickenings a Response to Environmental Stress?

*Miltoniopsis*, like other epiphytic orchids, uses numerous strategies that allow it to tolerate water stress, including having pseudobulbs for the storage of water. Its roots, which normally grow above ground or only partially in soil, are also prone to water stress, and like other epiphytic orchids, extensive phi thickenings can develop [23], structures that are typically rare or absent in terrestrial species [24]. These thickenings are thought to provide a mechanism for mechanical support during periods of water stress [22,24]. In tissue culture, *Miltoniopsis* orchids are grown on agar-solidified MS medium and we never observed any development of phi thickenings under these conditions. In horticultural settings, however, *Miltoniopsis* plants are usually grown in bark to allow good aeration and drainage, with the addition of sphagnum moss or perlite to maintain moisture levels, and the roots can develop phi thickenings. This is similar to wild cherry (*Prunus avium*), where phi thickenings were absent when grown in vitro, but developed quickly when plants were transferred to soil [14]. 

We investigated the induction of phi thickenings in phi thickening-free roots transferred from solid culture to four different media. Well-drained growth media containing fir bark or bark mixes resulted in phi thickening development even when regular watering was used, whereas more compact, water-retaining media (soil, sphagnum) only resulted in phi thickening induction under conditions of reduced watering. Our observations in *Miltoniopsis* orchids are consistent with observations of stress-induced phi thickenings in numerous other plant species. For example, loquat (*Eriobotrya japonica*) roots that underwent drought stress developed dramatic phi thickenings compared to normal conditions [19]. Our results are also consistent with the modified phi thickening production in *Cryptomeria japonica* seedlings in waterlogged soil. *Cryptomeria* roots, which normally have phi thickenings, showed a significant decrease in phi thickening production in waterlogged soil [20]. Similar studies on *Brassica oleracea* reported that salinity (80 mM NaCl in a hydroponics system) resulted in the development of phi thickenings in the inner cortex [12,16], and subsequent induction experiments have shown that both *B. oleracea* and *B. napus*, along with several other species within the Brassicaceae, will develop phi thickenings in the primary root when grown on agar plates containing either 80 mM NaCl or 1% (*w/v*) sucrose [17]. Several studies have also suggested that the formation of phi thickenings provides mechanical support for the root, which may aid penetration through the soil. For example, phi thickenings were present in *Ceratonia siliqua* when grown in soil, but were absent in perlite [34], indicating the effect of growth substrate. In maize, there is an increase in the formation of phi thickenings in the rhizodermis of the oldest portion of *Zea mays* roots grown in municipal solid-waste slag. This slag is dense, has a high pH, and is high in salt and heavy metals, and thus represents multiple abiotic stresses. Root growth in slag led to the formation of phi thickenings, but this was attributed to the roots’ need to overcome physical resistance caused by the dense slag [11].

## 4. Materials and Methods

### 4.1. Plant Material

Plants of *Miltoniopsis* cultivar Breathless were obtained from Dr. John Clemens of the Christchurch Botanical Gardens, and from Robertsons Orchids (West Woombye, QLD, Australia). Plants were maintained in a greenhouse, growing under natural light in standard fir bark-based orchid potting mix (either Southern Horticultural Products, Rolleston, New Zealand, or Osmocote Professional Orchid Mix, Scotts Australia, Bella Vista, NSW, Australia) with slow release fertiliser added every 4 months, with variations to these growth conditions described below. Tissue culture flasks containing *Miltoniopsis* cultivar Breathless were purchased from Woolf Orchid Culture (Drayton, QLD, Australia), and imported into New Zealand with the assistance of Tuckers Orchid Nursery (Auckland, New Zealand). Plants in culture were maintained in a temperature-controlled room at 23 °C with a 16 h light/8 h dark photoperiod using 36-watt white fluorescent tubes. Plants were grown in half-strength Murashige and Skoog medium (Sigma, St Louis, MO USA) with the addition of 3% (*w/v*) sucrose and 1% (*v/v*) plant preservative mixture (PPM) (Plant Cell Technology, Washington, DC, USA) to inhibit fungal contamination [35]. Plants were re-flasked every 3 months in this medium. Root material was harvested from plants grown in soil and on agar using a razor blade, and washed under running tap water to remove surface debris before being used in experiments.

### 4.2. Fungal Reinfection Experiments

Endomycorrhizal fungus was isolated from healthy soil-grown *Miltoniopsis* roots [36]. Roots were cut into segments, surface sterilised (0.5% (*w/v*) sodium hypochlorite, 2 min), and washed with sterile distilled water. Clumps of inner cortex cells were removed and macerated in a drop of sterile water, and covered with molten potato dextrose agar (DPA) (39 g/L, 55 °C) (Difco, Becton-Dickinson, Franklin Lakes, NJ, USA) poured over the cells. Plates were incubated in the dark at 23 °C until hyphae emerged from the agar, and then serially sub-cultured to obtain a pure culture. Slanting double cultures [37] were used for re-inoculation experiments. Flasks were poured containing basic MS medium in the bottom of the flask, with a layer of molten PDA medium carefully poured into each flask while it rested at 45° to create a slanting wedge of PDA medium on top of the MS medium. *Miltoniopsis* plants were sub-cultured on the MS medium while a disc of the fungal isolate was inoculated in the middle of the slanting PDA medium. Roots were excised and screened at various times after co-inoculation.

Pelotons and hyphae in orchid root cortical cells were stained with lactofuchsin [38]. Root sections were fixed in 3.7% (*v/v*) formaldehyde, 0.5% (*v/v*) glutaraldehyde, 0.1% (*v/v*) Triton X-100, and 0.1% (*v/v*) dimethyl sulfoxide in PME solution (50 mM Pipes, pH 7.2 (K^+^), 2 mM EGTA, 2 mM MgSO_4_) under vacuum for 2 h. After washing in PME, roots were stained in lactofuchsin (0.1% (*w/v*) acid fuchsin in 85% (*v/v*) lactic acid) for 2–3 h at 50 °C. Surface dye was removed by rinsing twice in de-staining solution (1:1:1; distilled water, 85% (*v/v*) lactic acid, glycerol), before roots were de-stained in this solution at 50 °C overnight, and before 50–100-μm-thick sections were cut with a Vibratome.

### 4.3. Microscopy

Observations of roots in phi thickening induction experiments were made by wide-field microscopy using a DMI6000 inverted microscope (Leica, Wetzlar, Germany) with 20× NA 0.7 and 63× NA 1.3 glycerol-immersion lenses, and photographed with a digital camera (model DFC310FX, Leica). Imaging was conducted using conventional transmitted light optics, polarised light optics, and with fluorescence using standard UV, blue, and green excitation filters.

The DMI6000 microscope was used as part of a confocal microscope system (model Leica SP5, Leica). Images were collected at high resolution (1024 × 1024 pixels) with three-fold line averaging, and with 1.00 μm step-size for Z-stacks of optical sections. Lignin autofluorescence and calcofluor white were both excited with 405 nm violet light, with emission collected from 420 to 480 nm. To prevent cross-talk between multiple fluorophores, the different fluorophores were excited sequentially in multiple labelling experiments. Concurrent transmitted light images were collected, either with conventional transmitted light optics or using polarised light settings.

Analysis of images was completed with ImageJ (FIJI installation, version 1.50, National Institutes of Health, Bethesda, MD, USA). All images were processed with Adobe Photoshop version CS4 (version 11.0.1, Adobe Systems, San Jose, CA, USA) using standard contrast and gamma tools.

### 4.4. Permeability of Phi Thickenings

The permeability of the phi thickenings in *Miltoniopsis* was tested through uptake experiments using the fluorescent tracer dye calcofluor white (Molecular Probes, Eugene, OR, USA), which, being membrane-impermeant, can only diffuse through the cell wall. Using published protocols [10], fresh roots were cut 2 cm from the root tip, washed under tap water, and dried with a paper towel. The cut end of the root and the root tip were sealed with Ados F2 glue (CRC, East Tamaki, New Zealand), which was water-resistant and had high bond strength to instantly bind to plant tissue to ensure that dye uptake occurred only through the apoplast, and a needle run several times on the root surface to rupture the velamen and let tracers into the cortex. Roots were incubated in 0.1% (*v/v*) calcofluor white or a water control for 18 h, rinsed with water, hand-sectioned, and immediately viewed with wide-field or confocal microscopy.

### 4.5. Phi Thickening Induction Experiments

As young plantlets growing in sterile culture on agar lacked phi thickenings, but developed thickenings some weeks after transplantation to standard orchid culture conditions, the control of phi thickening induction could be investigated. The development of phi thickenings was observed in roots with polarised light microscopy in hand-cut cross sections taken near the root tip, and at locations about 10–20 mm from the root tip. To investigate links with fungal infections, sterile tissue culture-grown *Miltoniopsis* plantlets were transferred to autoclaved tissue culture pots of fir, and placed in a temperature controlled room under sterile conditions at 23 °C with a 16 h light/8 h dark photoperiod using 36-watt white fluorescent tubes for 8 weeks. Observations of phi thickenings were made at 4, 6, and 8 weeks after transplantation.

To investigate the roles played by growth media and moisture, flasked plantlets were transplanted into sphagnum moss and incubated in a moisture chamber in the greenhouse for 4 weeks to acclimatise to the environment before further experiments. Twelve replicate plants were then transplanted into four different treatments; 100% fir bark, commercial orchid mix, a mixture of soil and fine potting mix, and a mixture of sphagnum moss and fir bark. Plants were grown in a shaded greenhouse with automated watering twice a week for 4 months, and excised roots checked for the development of phi thickenings. After 10 weeks, the effects of water stress were tested, with the watering of 10 plants from each treatment decreased to once a week, with two plants from each treatment watered twice a week as a control. The degree of water stress was evaluated through leaf conditions and colour.

## 5. Conclusions

Phi thickenings have been observed in more than 100 different plant species, including several monocots, diverse dicot families, and numerous gymnosperms [1,4]. That similar structures are present in so many diverse species suggests either that the phi thickenings have arisen multiple times through convergent evolution, or that they are an ancestral characteristic that has been conserved. In either case, the phi thickenings are likely to be functionally important for the roots. With phi thickenings having been observed in many agriculturally-important species, and with their formation and functionality often linked to abiotic stress, understanding the mechanisms through which they form might be agriculturally important. Our data have established that orchid root phi thickenings can be induced by a specific abiotic stress (water stress), and suggest that they function solely in stabilising root structure. The comparative ease with which orchids can be cultured, and the extensive nature of their phi thickenings, suggest that orchids may prove an ideal model system for understanding the pathways involved in phi thickening formation.
